# New developments for the Quest for Orthologs benchmark service

**DOI:** 10.1093/nargab/lqae167

**Published:** 2024-12-11

**Authors:** Adrian Altenhoff, Yannis Nevers, Vinh Tran, Dushyanth Jyothi, Maria Martin, Salvatore Cosentino, Sina Majidian, Marina Marcet-Houben, Diego Fuentes-Palacios, Emma Persson, Thomas Walsh, Odile Lecompte, Toni Gabaldón, Steven Kelly, Yanhui Hu, Wataru Iwasaki, Salvador Capella-Gutierrez, Christophe Dessimoz, Paul D Thomas, Ingo Ebersberger, Erik Sonnhammer

**Affiliations:** ETH Zurich, Department of Computer Science,Universitätstrasse 19, 8092 Zurich, Switzerland; SIB Swiss Institute of Bioinformatics, Quartier Sorge - Bâtiment Amphipôle, 1015 Lausanne, Switzerland; SIB Swiss Institute of Bioinformatics, Quartier Sorge - Bâtiment Amphipôle, 1015 Lausanne, Switzerland; Department of Computational Biology, University of Lausanne, Génopode, 1015 Lausanne, Switzerland; Applied Bioinformatics Group, Institute of Cell Biology and Neuroscience, Department of Biosciences, Goethe University, Max-von-Laue-Str. 13, D-60438 Frankfurt, Germany; European Molecular Biology Laboratory, European Bioinformatics Institute, Wellcome Genome Campus, Hinxton, Cambridge, Cambridgeshire CB10 1SD, UK; European Molecular Biology Laboratory, European Bioinformatics Institute, Wellcome Genome Campus, Hinxton, Cambridge, Cambridgeshire CB10 1SD, UK; Department of Integrated Biosciences, University of Tokyo, Tokyo 277-0882, Japan; SIB Swiss Institute of Bioinformatics, Quartier Sorge - Bâtiment Amphipôle, 1015 Lausanne, Switzerland; Department of Computational Biology, University of Lausanne, Génopode, 1015 Lausanne, Switzerland; Barcelona Supercomputing Center (BSC-CNS), Plaça d'Eusebi Güell, 1-3, 08034 Barcelona, Spain; Institute for Research in Biomedicine (IRB Barcelona), The Barcelona Institute of Science and Technology, Carrer Baldiri Reixac, 10, 08028 Barcelona, Spain; CIBER de Enfermedades Infecciosas, Instituto de Salud Carlos III, Monforte de Lemos, 3-5. Pabellón 11, 28029 Madrid, Spain; Barcelona Supercomputing Center (BSC-CNS), Plaça d'Eusebi Güell, 1-3, 08034 Barcelona, Spain; Institute for Research in Biomedicine (IRB Barcelona), The Barcelona Institute of Science and Technology, Carrer Baldiri Reixac, 10, 08028 Barcelona, Spain; Department of Biochemistry and Biophysics, Stockholm University, Science for Life Laboratory, Box 1031, SE-17121 Solna, Sweden; European Molecular Biology Laboratory, European Bioinformatics Institute, Wellcome Genome Campus, Hinxton, Cambridge, Cambridgeshire CB10 1SD, UK; Department of Computer Science, ICube, UMR 7357, Centre de Recherche en Biomédecine de Strasbourg, University of Strasbourg, CNRS, 1 rue Eugène Boeckel, 67000, Strasbourg, France; Barcelona Supercomputing Center (BSC-CNS), Plaça d'Eusebi Güell, 1-3, 08034 Barcelona, Spain; Institute for Research in Biomedicine (IRB Barcelona), The Barcelona Institute of Science and Technology, Carrer Baldiri Reixac, 10, 08028 Barcelona, Spain; CIBER de Enfermedades Infecciosas, Instituto de Salud Carlos III, Monforte de Lemos, 3-5. Pabellón 11, 28029 Madrid, Spain; Catalan Institution for Research and Advanced Studies (ICREA), Passeig de Lluís Companys, 23, 08003 Barcelona, Spain; Department of Biology, University of Oxford, South Parks Road, Oxford, OX1 3RB, UK; Department of Genetics, Harvard Medical School, Boston, MA 02115, USA; Department of Integrated Biosciences, University of Tokyo, Tokyo 277-0882, Japan; Barcelona Supercomputing Center (BSC-CNS), Plaça d'Eusebi Güell, 1-3, 08034 Barcelona, Spain; SIB Swiss Institute of Bioinformatics, Quartier Sorge - Bâtiment Amphipôle, 1015 Lausanne, Switzerland; Department of Computational Biology, University of Lausanne, Génopode, 1015 Lausanne, Switzerland; Department of Population and Public Health Sciences, University of Southern California, Los Angeles, CA 90033, USA; Applied Bioinformatics Group, Institute of Cell Biology and Neuroscience, Department of Biosciences, Goethe University, Max-von-Laue-Str. 13, D-60438 Frankfurt, Germany; Senckenberg Biodiversity and Climate Research Centre (S-BIK-F), Senckenberganlage 25, D-60325 Frankfurt am Main, Germany; LOEWE Centre for Translational Biodiversity Genomics (TBG), Senckenberganlage 25, D-60325 Frankfurt am Main, Germany; Department of Biochemistry and Biophysics, Stockholm University, Science for Life Laboratory, Box 1031, SE-17121 Solna, Sweden

## Abstract

The Quest for Orthologs (QfO) orthology benchmark service (https://orthology.benchmarkservice.org) hosts a wide range of standardized benchmarks for orthology inference evaluation. It is supported and maintained by the QfO consortium, and is used to gather ortholog predictions and to examine strengths and weaknesses of newly developed and existing orthology inference methods. The web server allows different inference methods to be compared in a standardized way using the same proteome data. The benchmark results are useful for developing new methods and can help researchers to guide their choice of orthology method for applications in comparative genomics and phylogenetic analysis. We here present a new release of the Orthology Benchmark Service with a new benchmark based on feature architecture similarity as well as updated reference proteomes. We further provide a meta-analysis of the public predictions from 18 different orthology assignment methods to reveal how they relate in terms of ortholog predictions and benchmark performance. These results can guide users of orthologs to the best suited method for their purpose.

## Graphical Abstract

**Graphical Abstract:**
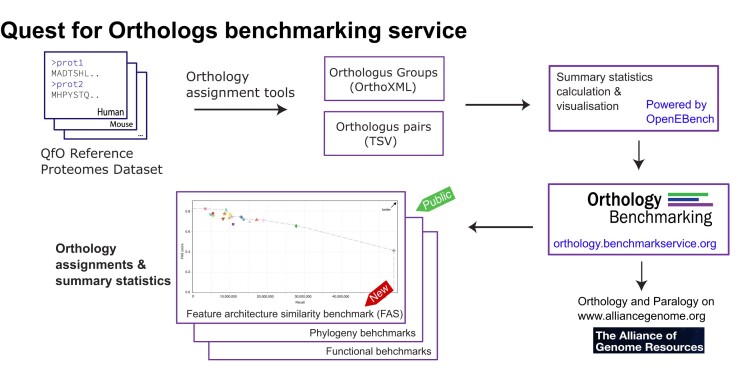

## Introduction

A central theme in evolutionary bioinformatics is the study of orthologs. Orthologs are genes or proteins with a shared genetic origin that have descended from one gene in their latest common ancestor species over the course of evolution, and are thus separated by a speciation event (1). This makes orthologs useful in several ways. Since they tend to have retained their function in different species, orthologs are often used for transferring functional information between species (2). This can, for example, accelerate our understanding of disease genes by studying their orthologs in model organisms. Furthermore, orthologs are valuable for phylogenetic studies since the complication of gene duplication is avoided.

The QfO benchmark service is one of the core resources that the Quest for Orthologs (QfO) consortium (3–5) provides to the evolutionary biology community. By standardizing the datasets and benchmarks, the resource makes it possible to compare orthologs predicted by different methods in a fair and unbiased way. The QfO benchmark service consists of a collection of benchmarks of different types to which developers of ortholog detection methods can submit their predictions on a predefined set of 78 reference proteomes from all domains of life. The selection of proteomes was made to be representative across all phyla, yet keeping the set small enough for computationally expensive methods to be run.

We here describe the latest developments of the orthology benchmark server. We have added a new benchmark based on the feature architecture similarity (FAS) method to measure the conservation of the architecture of features such as protein domains, transmembrane regions and disordered regions (6). The reference proteomes have been updated to ensure high accuracy of the sequences, and to be readily usable in other databases such as the Alliance of Genome Resources orthology resource (7). We further provide new modes of meta-analysis to globally compare the orthology inference methods in terms of their predictions and their benchmark performance.

## Results

### A new benchmark: feature architecture similarity

Most orthology assignment tools assume that orthologous sequences share the same evolutionary history over their entire length. This assumption finds its roots in the hypothesis that evolutionary constraints maintain the integrity of protein function, and consequently also of the architecture of protein domains conveying this function (8). Tree-based approaches assess the fulfillment of this assumption via the dominating evolutionary signal across the ortholog candidates. They accept an ortholog candidate, if the sequence tree reflects the evolutionary histories of the corresponding species. Graph-based approaches, in turn, investigate whether the pairwise distances between sequences justify the orthology assumption. Irrespective of the underlying concept, orthology assignment tools initially identify homologous sequences based on pairwise local sequence alignments. To reduce the computational burden of the orthology inference, but also the false-positive rate, only a subset of sequences with a significant local sequence similarity are propagated to the next analysis step. Since neither percent sequence similarity nor bit scores are reliable proxies of whether two sequences are orthologous, some tools test only candidates whose local alignment covers a predefined fraction *n* of positions from the longer sequence, where the default value of *n* is tool-specific (e.g., 0.5 in the case of InParanoid (9) or 0.61 in the case of Orthologous MAtrix (OMA) (10)). While such and similar filters are easy to devise and implement, their effects during the actual orthology inference are assessed, if at all, only during benchmarking the individual tools. Moreover, orthology assignments across larger evolutionary distances, for example, between eukaryotes and archaea where the latter tend to have shorter proteins (11), may benefit from a dynamic adjustment of the length cutoff rather than working with a fixed value (as, e.g., in OrthoFinder (12)). Eventually, domain gain and loss are relevant evolutionary mechanisms that modify the function of an evolutionarily old protein on individual evolutionary lineages. Tracing such changes may benefit from either no pre-filtering at all (13) or performing the orthology analysis on the domain level (14) as done in InParanoiDB 9 (15) and SonicParanoid2 (16).

**Figure 1.**
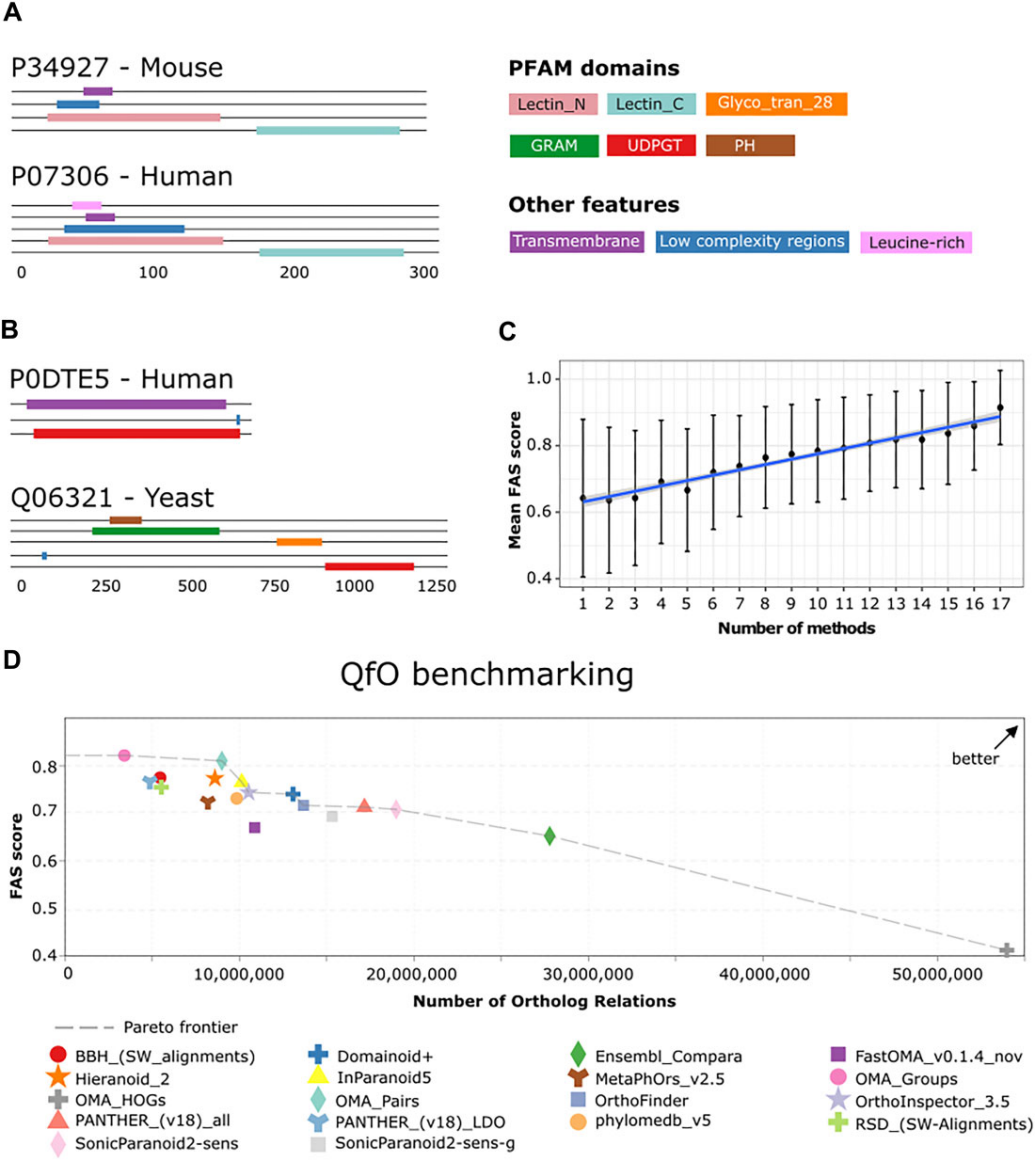
Feature architecture comparison as a novel benchmark in the QfO benchmark service. (**A**) Feature architecture comparison of an ortholog pair that was consistently found by all methods. The average bi-directional FAS score is 0.85 due to the Leucine-rich region that is present only in the human protein. (**B**) Feature architecture comparison of an ortholog pair that was assigned only by SonicParanoid. The two proteins differ substantially in both their length and in their feature architecture with the sole feature being shared is the C-terminal UDPGT Pfam domain. The average bi-directional FAS score is 0.43. (**C**) The correlation between the mean FAS score of protein pairs and the number of ortholog predictors supporting the orthology relationship (Pearson’s correlation coefficient: 0.98, *P* = 6e-12). (**D**) FAS benchmark performance versus the number of inferred orthologs for orthology assignment tools submitted to the latest QfO orthology benchmark service.

To shed light on how different orthology assignment tools cope with changing evolutionary histories along a sequence or with orthologs of substantially varying length, we introduce a new benchmark based on the pairwise comparison of protein feature architectures (6). In brief, the protein sequences of orthologous proteins are decorated with features, such as Pfam and SMART domains (17,18), signal peptides and transmembrane domains, and low complexity regions. The resulting multi-dimensional feature architectures are then compared between ortholog pairs predicted by the individual tools using, in turns, one of the two proteins as a reference (Figure 1A and B). The resulting similarity scores range between 0 (no shared feature) and 1 (the reference architecture matches a (sub-)architecture of the second protein) (6). In the benchmark, we assess for each tool the average bi-directional FAS scores across all predicted ortholog pairs.

We first investigated whether there is a dependency between the average bi-directional FAS score for a predicted ortholog pair and the number of orthology assignment tools that consistently support the orthology relationship. Figure 1C shows that both values are strongly positively correlated (Pearson’s correlation coefficient: 0.98, *P* = 6e-12). Ortholog pairs that are unanimously supported by all 18 methods have a mean bi-directional FAS score of >0.9. This value drops in a linear fashion to <0.7 for pairs supported only by one or two methods.

Individual tools tolerate differences in the feature architecture of orthologs to a varying extent. As a general trend, the average bi-directional FAS score decreases with increasing numbers of predicted orthology relations (Figure 1D). Ortholog pairs derived from OMA groups, which resemble cliques of orthologous sequences (see (10)), have the highest average FAS score with the lowest recall. OMA Hierarchical Orthologous Groups (HOGs), which result in about five-times more orthology relations, have by far the lowest average FAS score indicating that many of the related proteins differ substantially in their feature architectures. This likely is a consequence of considering many in-paralogous relations that arise by the hierarchical nature of the orthologous groups. The HOGs are rooted by a speciation event but then combine paralogous lineages that arose at a later time point in the course of the gene family evolution (10,19). Interestingly, this provides an indirect indication that feature architectures of paralogs tend to change more quickly than those of orthologs. The novel benchmark, however, reveals that some tools increase the number of orthology relationships substantially without sacrificing the FAS between orthologs. For example, compared to OrthoInspector 3_5, Domainoid + predicts ∼2.6 million additional orthology relationships (10.5 million versus 13.1 million) while the average FAS score drops only marginally by 0.003 units. Even more pronounced is the difference between both of these tools compared to FastOMA. While the number of FastOMA relationships is with 10.8 million only slightly higher than that of OrthoInspector 3_5 (but still considerably smaller than that of Domainoid+), the average FAS score is ∼0.1 units smaller than that of both OrthoInspector 3_5 and Domainoid +. This indicates considerable differences in the way FastOMA infers the orthology relationships (see section ‘Meta-analyses of public ortholog inference methods’ below). Furthermore, other tools including OrthoFinder, Panther-all, and Domainoid + are placed in the middle.

### New QfO reference proteomes (2022 dataset)

The QfO benchmarks are based on the QfO Reference dataset of proteomes containing the canonical protein sequences of every annotated protein-coding gene in a given species. This allows a standardized and fair benchmarking, and the results of individual inference methods can be directly compared. The QfO Reference Proteomes (https://www.ebi.ac.uk/reference_proteomes/) have been jointly designed for this task by the QfO consortium and UniProtKB (20), with a focus on including well-annotated species of medical and scientific interest, and on broadly covering the Tree of Life while staying of manageable size for every orthology inference provider. The dataset is updated annually; the version used in the present QfO benchmark (QfO Reference Proteomes 2022) comprises 78 species (48 Eukaryotes, 23 Bacteria and 7 Archaea) based on the UniProtKB 2022_02 release (apart from the *Danio rerio* [UP000000437] reference proteome from the 2022_03 release). In aggregate, this represents 1 383 730 protein sequences (988 778 canonical protein sequences and 394 952 isoforms).

The QfO Reference proteomes 2022 version has been improved in several ways compared to the previous version. The genome assemblies for six species have been updated to a newer version (Supplementary Table S1). The improved genome annotation of source databases (e.g., Ensembl and RefSeq) has been considered, as well as the manual curation of entries in UniProtKB. In individual cases, e.g., *Physcomitrium patens*, this affected more than half of the proteins in the reference proteome. The resulting Reference Proteomes therefore not only represent a common basis for the software benchmark, but the orthology assignments remain an up-to-date resource also for applied analyses investigating the evolution of protein-coding genes (see section ‘Data reuse by the Alliance of Genome Resources’ below). The QfO Reference Proteomes are available for download in various formats: the protein sequences as FASTA and SeqXML files, CDS sequences for most proteins as FASTA files, and, for an increasing number of species, genomic locus coordinates are available in the XML format.

Reference proteome datasets are generated using a gene-centric approach which identifies all protein isoforms for a gene and selects the canonical protein sequence as representative of the set. The generation of these datasets requires a synchronized update effort of the underlying databases that are the source of protein sequences and gene annotations (including the European Nucleotide Archive, Ensembl, RefSeq and Model Organism Databases). We continuously monitor for improved annotations, incorporating feedback from the scientific community. One example is *Xenopus tropicalis*: in the 2020_04 UniProt release, we incorporated the latest annotations (GCA_000004195.4) from the Ensembl Rapid release, while in the 2022_02 release, we integrated annotations from RefSeq, which overall increased the similarity of the *X. tropicalis* proteins to their orthologs in *Xenopus laevis* (6). Similarly, for *Danio rerio*, we transitioned from using Ensembl to the latest RefSeq annotation as recommended by the ZFIN community, resulting in a higher predicted gene count and subsequently increasing the number of canonical sequences from 25 698 to 26 355. This update was integrated into the 2022_03 UniProt release and QfO 2022 release.

To help identify such changes in reference proteomes, we continue to provide STATS files (Supplementary Table S2). This file includes a summary of changes to the number of records in the canonical FASTA, additional FASTA and gene symbol to UniProt accession (gene2acc) mapping files, along with a report of changes to the source genome assembly for a proteome. This helps to easily identify any drastic changes in numbers for a given species and also to track changes over longer periods of time.

### Meta-analyses of public ortholog inference methods

For benchmarking purposes, method developers are requested to provide all the orthologous pairs inferred by their own methods using the QfO reference proteome dataset. These pairs are made freely available under the FAIR principle through the OpenEBench platform. They then become one of the data sources for the DIOPT (21) orthology metapredictor and the Alliance of Genome Resources orthology resource (7). Because all methods use the same reference proteomes, these pairs are also a valuable dataset for analyzing how different inference methods relate to each other. We provided such an analysis in our report of the previous release of the benchmarking service (5). Since we believe this is a unique lens under which to behold new orthology inference methods or new versions of existing tools, we repeated the analysis for the latest version.

**Figure 2.**
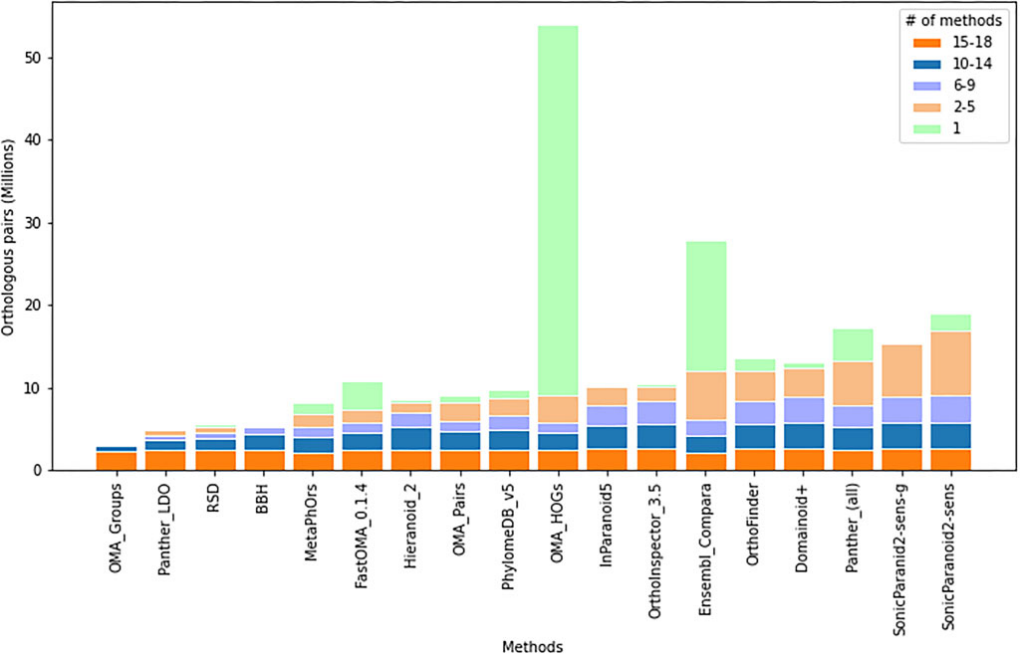
Orthologous pairs inferred by the 18 public methods in the benchmarking service. Subsections of the bars represent the number of methods that share the same pairs, including the method in question. Green parts of the bars are unique to the method. Methods are ranked by the number of pairs they share with at least one other method (non-green part of the stacked bars).

Figure 2 shows what proportion of each method’s predictions is shared with other orthology inference methods. Most methods tend to predict pairs that are also predicted by at least one other method, and this stays true both when including all the methods in the comparisons or selecting only one method of a redundant set of methods (e.g., including only one of the SonicParanoid predictions). In this comparison, as was the case in the previous release (QFO 2020 release), a few methods stand out by predicting a relatively low number of pairs, but which are highly congruent with the other methods: OMA Groups, Panther LDO and the most classical methods — Bidirectional-Best-Hit (BBH) and Reciprocal Shortest Distance (RSD). All of these methods have in common to aim mainly at inferring 1-to-1 orthologs relations and be highly sensitive at the expense of specificity. On the other hand, Ensembl Compara and OMA HOGs predict a vast amount of orthologous pairs, of which most are not shared with other methods. FastOMA, one of the two new inference methods in this benchmark release, is joining most ‘balanced’ methods in predicting many pairs in common with other methods with a moderate number of unique predictions. SonicParanoid2 is the other new addition to this benchmark. It predicts a higher number of pairs than most methods (except the two outliers mentioned below), including the highest proportion of pairs predicted by at least one other method. Note that this is true even when only considering a non-redundant set of orthology predictions (Supplementary Figure S1).

**Figure 3.**
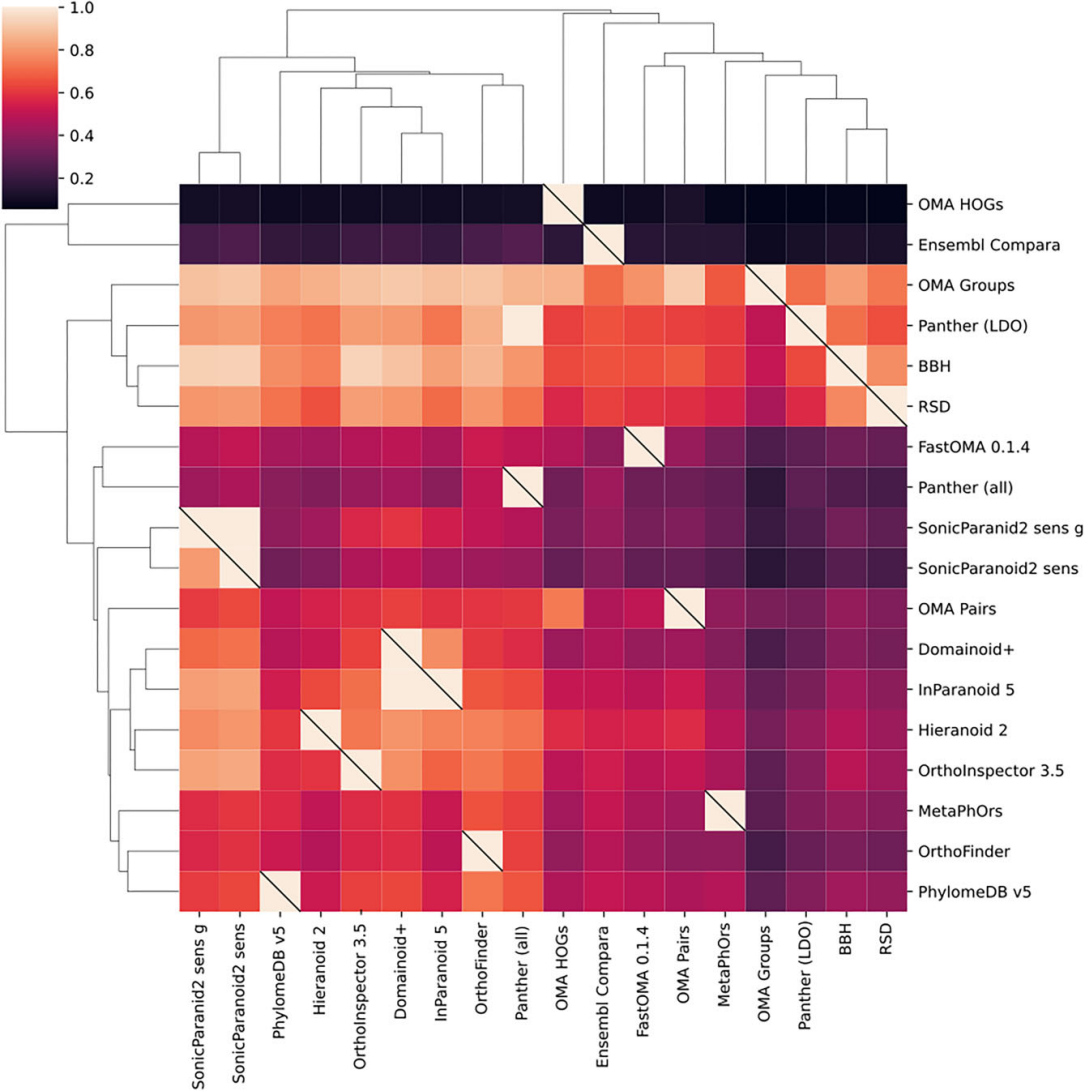
Pairwise representation of overlap between orthology inference methods included in the QfO Benchmarking service. The heatmap shows the proportion of the pairs inferred by methods on the right side that are found by methods on the bottom. The heatmap is hierarchically clustered on rows and columns by similarity with the corresponding trees shown.

We then performed pairwise comparisons between methods to analyze how much they overlap individually (Figure 3). As previously seen, there is overall only moderate similarity between methods, with an average overlap of 0.53. However, some of the methods have an overlap of 1 to another one. This indicates one method predicting a subset of the other and concerns only predictions uploaded by the same method developers. These include SonicParanoid2-sens-g pairs as a subset of SonicParanoid2-sens pairs, Inparanoid5 pairs as a subset of Domainoid + pairs and Panther_LOD as a subset of Panther pairs. Contrary to the previous release however, there is now a limited overlap between PhylomeDB and MetaPhOrs, which used to be subsets. MetaPhOrs is a meta method that joins different orthology predictions into a single prediction, which is computationally very expensive and hampers its update. Due to high computational costs and green computing principles, the MetaPhOrs predictions submitted in this version of the QfO benchmark were not based on a recomputation of the database with the new proteomes but rather the result of tracing back the new predictions submitted for QfO to the existing MetaPhOrs database, which includes all QfO species. This resulted in a substantial loss of orthologous pairs predictions which could explain the lower overlap between PhylomeDB and MetaPhOrs and underscores the difficulty of tracing records across genome annotations.

It is interesting to note that the results of the newly added FastOMA do not have high overlap with other OMA predictions which indicates that the difference in methodology between this new method and its predecessors is substantial. This is further corroborated by the observation that the average FAS score of the ortholog pairs assigned by FastOMA are substantially smaller than that of other orthology assignment tools with a comparable number of assigned pairs (see Figure 1). OMA standalone is based on all-against-all protein sequence comparison while FastOMA is based on a preliminary round of k-mer-based clustering in pre-existing gene families followed by gene tree based orthology inference (22). FastOMA does use prediction from the OMA Standalone algorithm as source of its initial gene families, which is the only relationship between these methods but our results indicate this has a limited effect on the similarity of these predictions.

**Figure 4.**
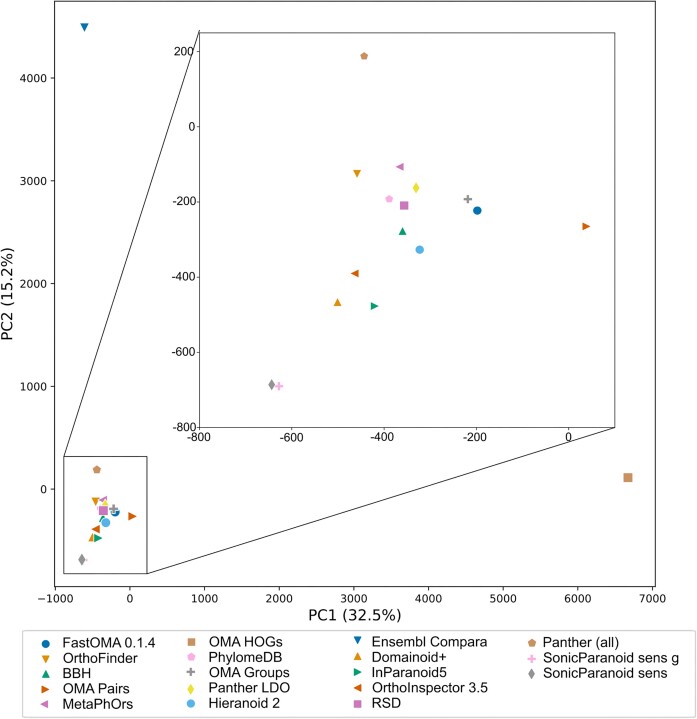
Relatedness of orthologous pair predictions between the orthology inference methods included in the QfO Benchmarking service by PCA. The inset shows an enlarged picture of the cluster that contains all methods except Ensembl Compara and OMA HOGs.

**Figure 5.**
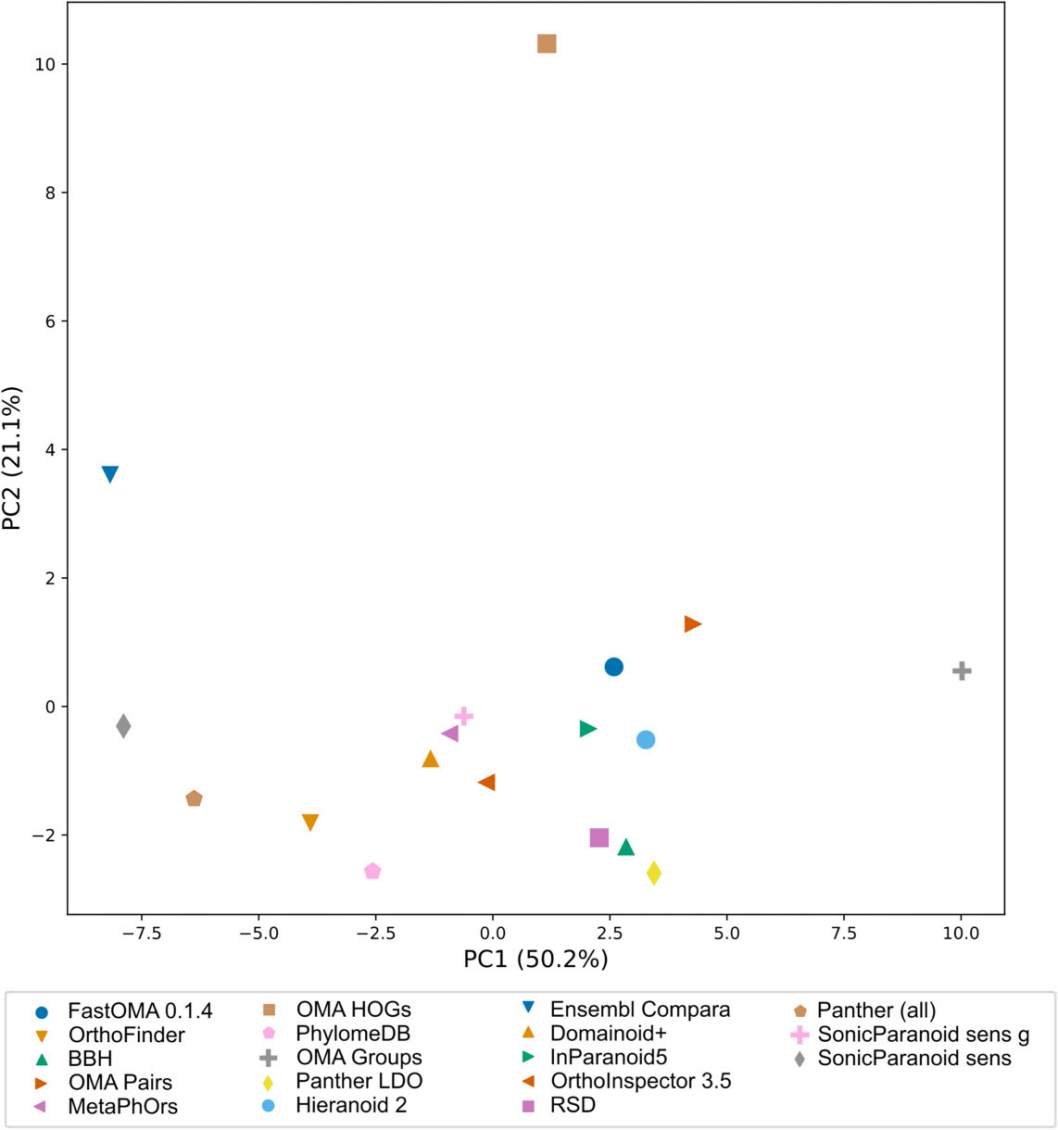
Relatedness of benchmark performance between the orthology inference methods included in the QfO Benchmarking service by PCA.

Another way to show relatedness between the orthology inference methods is by principal component analysis (PCA). We generated 2D plots of the first two components from binary vectors representing orthologous pair predictions (each column representing the prediction, or not, of a pair by each method) (Figure 4) and for all the benchmark results (Figure 5). In common for both these plots is that Ensembl Compara and OMA HOGs are clear outliers, which is likely due to their high numbers of unique predictions (Figure 2). Substantial differences exist, however, for instance in the ortholog pairs plot (Figure 4) the two SonicParanoid2 methods are outliers and very close to each other, but in the benchmark results plot (Figure 5), only SonicParanoid2-sens is an outlier while SonicParanoid2-sens-g is placed very centrally. This indicates that despite strong similarity in ortholog predictions, such as one being a subset of the other, two methods can perform differently in the benchmarks.

To examine the similarities and differences between orthologous pairs found by different tools, we explored their species distribution. First, we note that many of the pairs that are inferred by all methods are vertebrate proteins. Human and mouse, in particular, have ∼75% of their proteome involved in such ‘unanimous’ pairs. Only other vertebrate species (*Gorilla gorilla*, *Rattus norvegicus*, *Lepisosteus oculatus*, *Pan troglodytes*, *Canis lupus*, *Bos taurus* and*Monodelphis domestica*) have more than half of their proteomes covered by such pairs. This is likely due to the fact that the QfO Reference Proteome dataset is rich in closely related vertebrate species and thus orthology calling is a less challenging task. At the other end of the spectrum, the proteomes of *Zea mays* and *Physcomitrella patens* have <1% of their proteomes involved in a ‘unanimous’ pair—likely resulting from their genomes having experienced Whole Genome Duplications. Paralogy is not explicitly handled by the most basic methods in this benchmark (RBH and RSD) and generally introduce difficulty in orthology calling.

### Data reuse by the Alliance of Genome Resources

**Figure 6.**
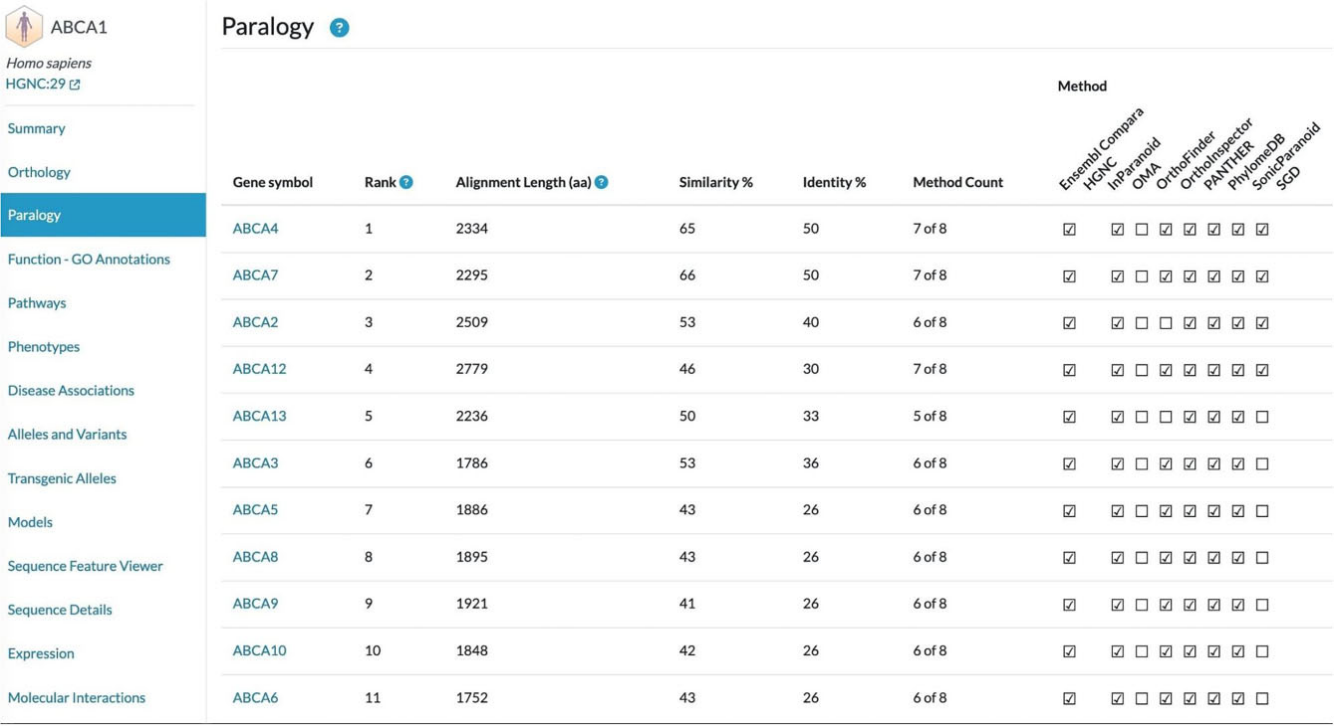
Paralogs of human protein ABCA1, as shown on the Alliance of Genome Resources website. Paralogs are ordered by protein sequence similarity (including pairwise alignment length and % amino acid similarity) as well as agreement across different QfO member resources. The URL for this specific table is https://www.alliancegenome.org/gene/HGNC:29#paralogy.

The Alliance of Genome Resources (Alliance) continues to use orthologs predicted by QfO member resources. Recently, the Alliance has also made within-species paralogs available (7). Users had requested this feature to help identify genes that may partially complement each other functionally, which can be important for interpreting genetic loss-of-function studies. Similarly to how orthologs are treated in the Alliance data and website, paralogs are obtained by integrating predictions from different QfO member resources into the Drosophila Research and Screening Center (DRSC) Integrative Ortholog Prediction Tool (DIOPT) version 9.1 developed by the DRSC (21,23). Currently, the Alliance paralogs are calculated using the 2020 benchmarking set of reference proteomes provided by UniProt (20). The paralog information is downloaded directly from the QfO benchmarking website when available (OMA (24,25) and PANTHER (26)). Paralogs from additional QfO methods were obtained either directly from those resources (Ensembl Compara (27,28) and PhylomeDB (29)), or calculated locally, for the same (2020) UniProt reference proteomes release (Inparanoid (15), OrthoFinder (12), OrthoInspector (30) and SonicParanoid (16)). In addition, this paralog assembly also included the manually curated *Saccharomyces cerevisiae* paralog pairs (31) from SGD (Saccharomyces Genome Database). Within-species paralogs can now be browsed on the Alliance website (alliancegenome.org), for any selected gene. Figure 6 shows the Paralogy section of an Alliance page for an example, the human ABCA1 protein.

## Discussion

The QfO benchmark service is a central resource for the orthology community, and its continued updating and development are important to both providers and users of orthology information. We here present the incorporation of the new FAS benchmark which is a welcome addition to the other seven benchmarks. Two new ortholog prediction methods are included in this update, which was used to analyze how different methods are related to each other.

The present dataset of QfO reference proteomes comprises 78 species, which have been selected by the community to cover all domains of life. Compared to the vast amount of complete proteomes now available this is a very small number, and coverage of some clades may not be optimal. However, any additional proteomes would increase the already heavy computational burden of the benchmarking as well as the generation of the ortholog predictions; hence, a balanced strategy to improve coverage is to replace redundant proteomes with less redundant ones. This way, we can keep the service more accessible to developers of new orthology inference methods.

Each benchmark provides the performance of all methods in terms of proxies for recall and precision. It is tempting to combine these measures in order to obtain a single performance measure that could be used to rank the methods, but because the methods have very different tradeoffs between recall and precision, deciding which method is the best depends on which aspect is considered most important. While each benchmark plot is equipped with a coarse grouping of the methods into four groups based on quartiles or clustering, these do not necessarily reflect true optimality. Instead one can look at local optimality in terms of placement on the Pareto frontier, where the locally best method ‘shadows’ other methods. This approach however also has potential issues, especially for summary statistics, for instance that being on the Pareto frontier is only a yes or no score, yet a method may be very close but not on the frontier.

The current benchmark suite is built around full protein orthology assignments, but as mentioned above in the FAS section, domain architecture may change during evolution which can cause changes in function. If the evolutionary event involves recombination of domains, this can lead to inconsistent or discordant orthology relationships, where different domains on the same protein have different evolutionary histories (14,32). In such cases of partial orthology, aiming for full-length protein orthology will inevitably miss some orthologous relationships. A possible remedy could be to devise a domain-oriented benchmark, but this will only be as good as current domain annotations, which do not capture all possible domain configurations. One could see the domain parsing itself as part of the challenge, but it would require redesigning the benchmarking pipeline to handle freely defined subsequences, and likely it could only be done for species discordance benchmarks since the other benchmarks rely on full-length protein annotations.

## Supplementary Material

lqae167_Supplemental_File

## Data Availability

The used proteome data are available at https://ftp.ebi.ac.uk/pub/databases/reference_proteomes/previous_releases/qfo_release-2022_02_with_updated_UP000000437/QfO_release_2022_02_with_updated_UP000000437.tar.gz. The predicted ortholog data are available at https://orthology.benchmarkservice.org/proxy/projects/2022/. These links are also found at https://orthology.benchmarkservice.org/.

## Notes

The member list of the Quest for Orthologs Consortium is provided in the Acknowledgments section.
