# Supplementary material for: New developments for the Quest for Orthologs benchmark service
Source: NAR Genom Bioinform. 2024 Dec 11;6(4):lqae167. doi: 10.1093/nargab/lqae167 (PMC11632614; doi:10.1093/nargab/lqae167)
Supplement: lqae167_Supplemental_File [file lqae167_supplemental_file.pdf]

Supplementary Table 1. Assembly updates in QfO Reference Proteomes. The proteomes for which the reference assembly changed from the last benchmark release are listed together with the corresponding assembly versions and %change in total number of sequences.

Proteome UPID	Organism	UniProtKB 2020_04	UniProtKB 2022_03	%change in sequences
UP000000437	Danio rerio (Zebrafish)	GCA_000002035.4 Ensembl	GCF_000002035.6 Refseq	-0.2%
UP000002254	Canis lupus familiaris (Dog)	GCA_000002285.2 Ensembl	GCA_000002285.4 Ensembl	-3.8%
UP000002494	Rattus norvegicus (Rat)	GCA_000001895.4 Ensembl	GCA_015227675.2 Ensembl	51%
UP000006727	Physcomitrium patens (Spreading earthmoss)	GCA_000002425.2 ENA/EMBL	GCA_000002425.2 EnsemblPlants	54%
UP000007305	Zea mays (Maize)	GCA_000005005.6 EnsemblPlants	GCA_902167145.1 EnsemblPlants	-36%
UP000008143	Xenopus tropicalis (Western clawed frog)	GCA_000004195.4 Ensembl	GCF_000004195.4 Refseq	-31%

Supplementary Table 2. STATS file for *Danio rerio* (2022 Release). The STATS file reports change in number of entries in proteomes between releases, as well as differences in assembly and provider

Proteome_ID	UP000000437
NCBI Taxonomy _ID	7955
#(1) Number of entries in main fasta (canonical) [last QfO release number of entries, difference between current and last number of entries, percent change between current and last release]	26355 (prev:25706, diff:649, %change:3)
#(2) Number of entries in additional fasta (isoforms) [last QfO release number of entries, difference between current and last number of entries, percent change between current and last release]	20591 (prev:21382, diff:-791, %change:-4)
#(3) Number of entries in gene2acc mapping file [last QfO release number of entries, difference between current and last number of entries, percent change between current and last release]	46946 (prev:47570, diff:-6<br>24, %change:-1)
Assembly(Previous_Assembly)	GCF_000002035.6 (GCA_000002035.4)
Source(Previous_Source)	Refseq (Ensembl)
Species_Name	Danio rerio (Zebrafish) (Brachydanio rerio)

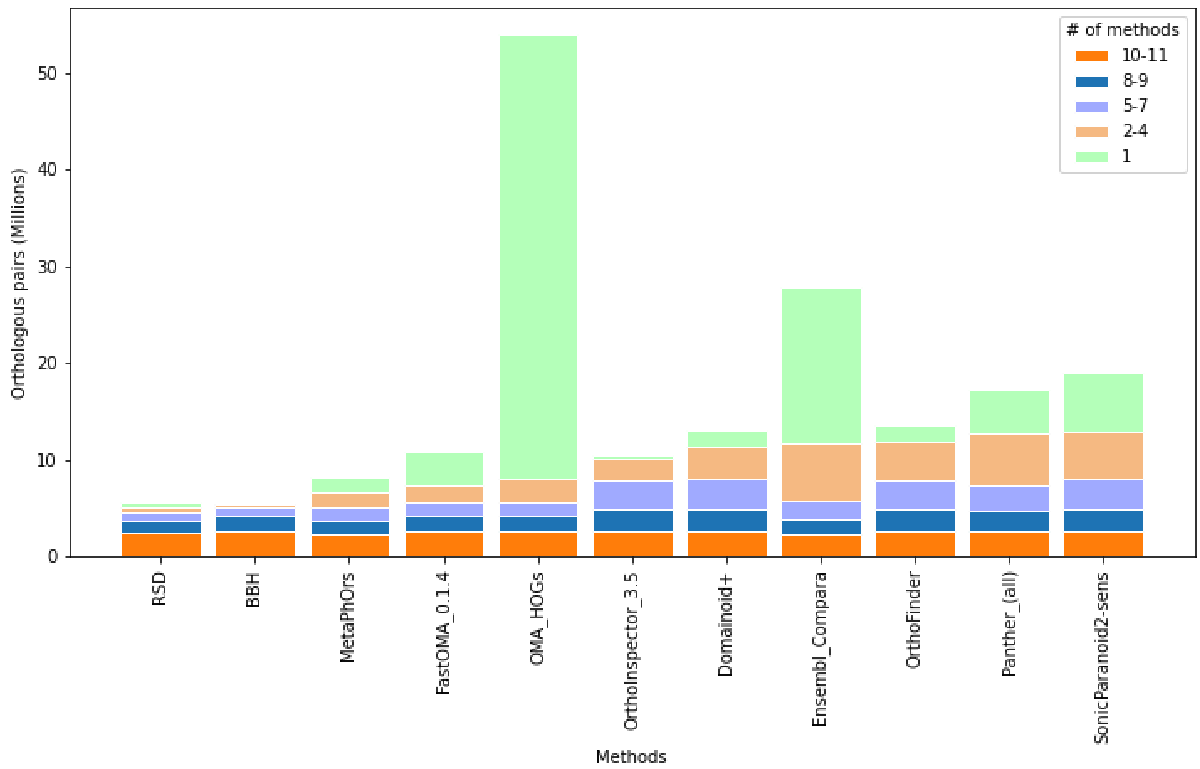

Supplementary Figure 1. Orthologous pairs inferred by 11 of the 18 public methods, in the benchmarking service, in millions. It is similar to Figure 2 but removes redundancy between included methods by selecting only one method in a group of methods with the same algorithmic underpinning, such as OMA, SonicParanoid 2, etc.. Subsections of the bars represent the number of methods that share the same pairs, including the method in question. Green parts of the bars are unique to the method. Methods are ranked by the number of pairs they share with at least one other method (non-green part of the stacked bars).
